# Insulin and Autophagy in Neurodegeneration

**DOI:** 10.3389/fnins.2019.00491

**Published:** 2019-05-22

**Authors:** Natália Prudente de Mello, Ana Maria Orellana, Caio Henrique Mazucanti, Geovanni de Morais Lima, Cristoforo Scavone, Elisa Mitiko Kawamoto

**Affiliations:** ^1^Laboratory of Molecular and Functional Neurobiology, Department of Pharmacology, Institute of Biomedical Sciences, University of São Paulo, São Paulo, Brazil; ^2^Laboratory of Molecular Neuropharmacology, Department of Pharmacology, Institute of Biomedical Sciences, University of São Paulo, São Paulo, Brazil

**Keywords:** obesity, type 2 diabetes, insulin, autophagy, neurodegenerative diseases

## Abstract

Crosstalk in the pathophysiological processes underpinning metabolic diseases and neurodegenerative disorders have been the subject of extensive investigation, in which insulin signaling and autophagy impairment demonstrate to be a common factor in both conditions. Although it is still somewhat conflicting, pharmacological and genetic strategies that regulate these pathways may be a promising approach for aggregate protein clearancing and consequently the delaying of onset or progression of the disease. However, as the response due to this modulation seems to be time-dependent, finding the right regulation of autophagy may be a potential target for drug development for neurodegenerative diseases. In this way, this review focuses on the role of insulin signaling/resistance and autophagy in some neurodegenerative diseases, discussing pharmacological and non-pharmacological interventions in these diseases.

## Introduction

Metabolic disorders such as obesity and type 2 diabetes (T2D) are a worldwide epidemic challenge of the 21st century that, besides resulting in reduced life expectancy and increased medical comorbidities, have been said to be risk factors for the development of neurodegenerative diseases (El Sayed et al., 2012). According to literature, both obesity and T2D contribute to an increased prevalence of dementia and cognitive decline (Arvanitakis et al., 2004; Kopf and Frölich, 2009; Nameni et al., 2017; Zhang et al., 2017), as well as to the onset of insulin resistance (Schneeberger et al., 2015), which is proposed to play a critical role in the link between metabolic disorders and central nervous system (CNS) impairment (Schneeberger et al., 2015; Nameni et al., 2017). Nowadays, it is known that insulin receptor (IR) is widely expressed in the CNS, and, although the exact link between brain insulin and neurodegenerative diseases remains still unknown, a plethora of studies have demonstrated that an optimal insulin signaling homeostasis is important to the maintenance of brain health.

The maintenance of cellular homeostasis is a complex process, which includes an interesting crosstalk between autophagy and apoptosis, involving, among other processes, the regulation of the phosphatidylinositol (3,4,5)-trisphosphate kinase (PI3K)-protein kinase B (AKT)-mammalian target of rapamycin (mTOR) and AMP-dependent protein kinase (AMPK) pathways. The mTOR is a serine/threonine kinase that is ubiquitously expressed, modulating cell proliferation, protein synthesis, and death or survival signalings. Localized mainly in the cytoplasm, mTOR is composed of two different protein complexes, mTOR complex 1 (mTORC1) and mTOR complex 2 (mTORC2), which differ in many respects, including protein complex components, and responsiveness to rapamycin, as well as activators or downstream signaling pathways. However, crosstalk between both complexes is mediated by AKT (Perluigi et al., 2015). The AKT signaling pathway can be triggered by insulin-like growth factor 1 (IGF-1) and insulin, which bind to transmembrane receptors and activate insulin receptor substrate 1 (IRS-1), leading to PI3K activation and subsequent phosphatidylinositol (3,4,5)-trisphosphate (PIP_3_) generation. AKT is responsible for the inhibition of negative regulators of mTOR, such as tuberous sclerosis complex (TSC1/2), whilst pyruvate dehydrogenase kinase 1(PDK1) induces the mTOR activator Rheb (Ras homolog enriched in brain), thereby releasing the mTOR complex to phosphorylate its targets (Perluigi et al., 2015). Interestingly, the chronically hyperactivation of mTOR by hyperinsulinemia is proposed to contribute to insulin resistance (Ueno et al., 2005).

**FIGURE 1:**
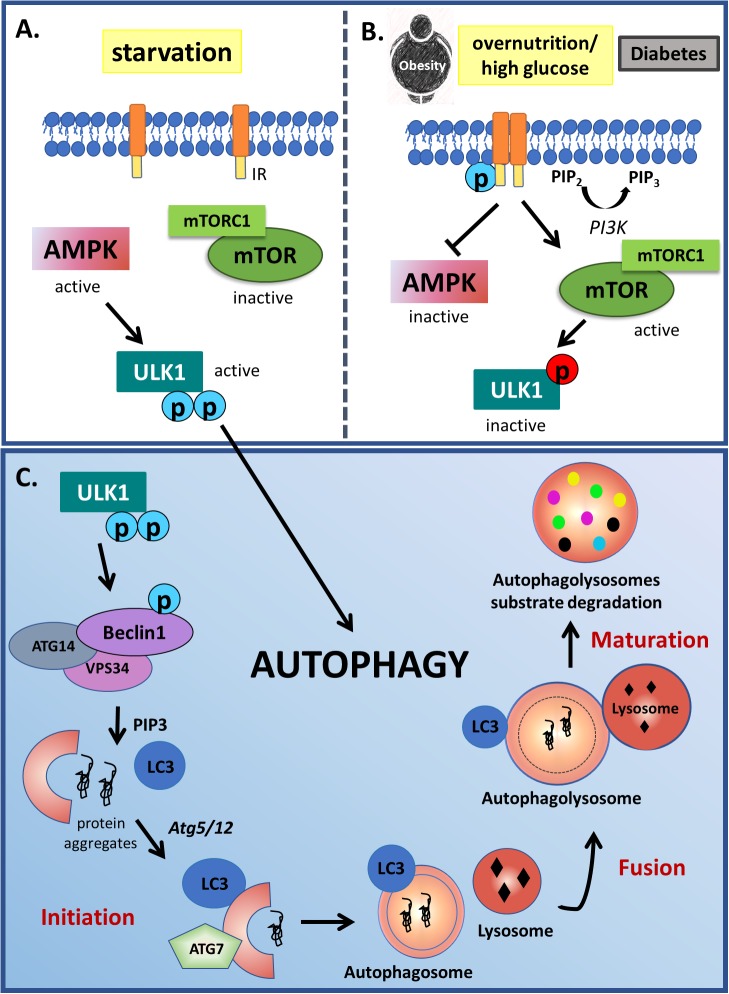
Autophagy modulation by starvation and insulin signaling. **(A)** Autophagy is classically activated in starvation conditions through ULK1 activity. In low nutrient conditions, AMPK signaling is activated, phosphorylating ULK1 in two different activation sites. Opposite to starvation, **(B)** in overnutrition condition or diabetes, PI3K type I is activated by growth factor receptors resulting in mTOR activation and AMPK inactivation. mTOR complex TORC1 phosphorylates and inactivates ULK1 (red circles), which is an initiator of mammalian autophagy. **(C)** During this process, protein-prone aggregates or damaged organelles are engulfed by double membrane structures called autophagosomes, which fuse with lysosomes or vacuoles allowing the degradation of their cargo material in the autophagolysosomes. This complex process starts with ULK1 phosphorylation in 2 different activation residues (indicated by blue circles). Active ULK1 phosphorylates Beclin 1, that together with other components induces the formation of phagophore, an initial membrane component of the isolation membrane, which then expands, curves and closes due to two conjugation pathways dependent of ATG5-ATG12 action to LC3 lipidation, turning into a completed autophagosome.

Autophagy is an important cellular process, whereby cell debris, including proteins and organelles, are degraded and recycled in order to prevent the build up of protein clusters and aggregates (Klionsky and Emr, 2000; Rubinsztein et al., 2005). During this process, portions of cytoplasm or organelles are engulfed by double membrane structures called autophagosomes, which fuse with lysosomes or vacuoles allowing the degradation of their cargo material (organelles and macromolecules) and its subsequent release into the cytosol for recycling (Kundu and Thompson, 2005). This complex process is regulated by more than 30 autophagy-related proteins (ATGs), as well as by the PI3K, the Unc-51-like kinase 1 (ULK1) and the microtubule-associated protein 1 light chain 3 (LC3) pathways. Under physiological condition, PI3K type I is activated by growth factors resulting in mTOR activation and consequently autophagy downregulation by phosphorylating and inactivating ULK1, which is an initiator of mammalian autophagy (Axe et al., 2008). Besides mTOR, AMPK also suppresses autophagy through the phosphorylation of different amino acid residues of ULK1 (Nishida et al., 2009). However, under some stress condition such as nutrient starvation, ULK1 is dephosphorylated by protein phosphatase 2A (PP2A), resulting in phosphorylation of multiple substrates and initiation of the conventional autophagy (Axe et al., 2008; Nishida et al., 2009; Mizushima and Komatsu, 2011). After that, together with other components, the Beclin1-containing class-III PI3K complex (Beclin 1, VPS15 and ATG14, class III VPS34) induces the formation of phagophore, an initial membrane component of the isolation membrane, which then expands, curves and closes due to two conjugation pathways, the ATG5-ATG12 and the LC3, turning into a completed autophagosome (Mizushima and Komatsu, 2011; Figure 1).

In this way, this review summarizes the origin and the role of insulin in the CNS, and discusses the relationship between insulin and autophagy in some neurodegenerative diseases, such as Alzheimer’s disease (AD), Parkinson’s disease (PD), Huntington’s disease (HD), amyotrophic lateral sclerosis (ALS) and frontotemporal dementia (FTD), as well as in spinocerebellar ataxia type 3 (SCA3) and Lafora disease (LD). Although there are at least three well-known distinct autophagic pathways, such as chaperone-mediated autophagy, microautophagy and macroautophagy, this review focuses on macroautophagy, hereafter referred to as autophagy.

## Insulin Resistance in the Central Nervous System and Neurodegeneration

The sugar lowering properties of the endocrine secretion of the pancreas were described by Banting and Best (1922), and insulin itself was crystallized shortly after, in 1926 (Abel, 1926). The number of scientific publications describing insulin and its functions exploded in the 1940s and, until the 1960s, most of the scientific community considered the brain to be an organ insensitive to insulin. The first half of 60s, however, was punctuated with compelling work done by separate groups that forced this discussion to be reopened. Work published in 1961 by Rafaelsen showed glucose uptake modulation by insulin in isolated rat spinal cord (Rafaelsen, 1961a) as well as rat cortical brain slices and cerebellum (Rafaelsen, 1961b). Butterfield et al. (1966), after measuring brain glucose uptake in 5 healthy male volunteers with insulin infusions, suggested that the human brain could be, after all, sensitive to insulin (Butterfield et al., 1966).

Recent work has confirmed insulin effects in the brain, although these can be quite distinct from its effects in the periphery, although the idea that insulin has exclusively neurotrophic effects in the brain has been contested by a few recent publications. In 2016, García-Cáceres et al. (2016) showed that signaling through the IR in hypothalamic astrocytes may affect the activity of glucose-sensing pro-opiomelanocortin neurons, suggesting the importance of insulin in the regulation of brain glucose uptake (García-Cáceres et al., 2016). Insulin-like growth factor I (IGF-I) is another important endogenous ligand of the IR, and works alongside insulin binding to the astrocytic IR in order to activate a non-canonical pathway, the mitogen-activated protein kinases (MAPK)/protein kinase D (PKD) pathway, which ultimately leads to the membrane translocation of glucose transporter (GLUT)1, a glucose transporter previously known to be insulin-insensitive. The combined effects of insulin and IGF-1 therefore modulate brain glucose (Fernandez et al., 2017). The following sections focus on the neurotrophic effects of insulin.

## Origins of Brain Insulin

Insulin was first successfully identified in dog cerebrospinal fluid (CSF) in 1967 by Margolis and Altszuler (1967). Through a continuous intravenous insulin infusion and periodic CSF and blood sampling, Margolis & Altszuler showed, for the first time, that fasted levels of insulin in the CSF were, on average, 27% of the insulin concentration found in plasma. Furthermore, it was the first report proving that insulin could cross the brain barriers, albeit in a very limited way. The concept that insulin can cross the blood-brain and the blood-CSF through a saturable transport system, and not by passive diffusion, would then become a significant milestone that is still regarded as the most accepted hypothesis describing insulin within the brain.

Although pancreas is, undoubtedly, the body’s primary source of insulin, the idea that insulin found in brain tissues and CSF is exclusively derived from the blood was first challenged in 1978 by Havrankova et al. (1978), in a paper showing high levels of insulin extracted from different brain regions, with the highest concentrations being found in the olfactory bulb and hypothalamus. Following this observation, the same group confirmed their findings in models of obese hyperinsulinemic and diabetic insulinopenic rats. In contrast to what was observed (and expected) in the liver, IR levels throughout the brain did not change in either experimental group, suggesting the participation of the brain barriers in the protection of the CNS from dramatic peripheral insulin oscillations. Furthermore, central insulin levels also did not change in any of the tested models, suggesting *de novo* synthesis of the hormone in the brain (Havrankova et al., 1979).

Central insulin biosynthesis outside the hypothalamus is still controversial. Observations of insulin production in primary neuronal cell cultures were first reported in 1986 by Clarke et al. (1986). Analyzing the released media from whole-brain primary neuronal cultures, they showed by radioimmunoassay and HPLC analysis not only the presence of secreted insulin but also its positive regulation by depolarization, via K^+^ and Ca^2+^. As proof that these observations were specific to neuronal depolarization, Clarke showed that no such effect was possible in glial cells in culture. Preproinsulin mRNA and protein was reported in pyramidal neuronal cells of the hippocampus and olfactory bulb (Kuwabara et al., 2011), with further studies showing extensive distribution of insulin expression throughout the brain, with higher levels in the hippocampus, striatum, thalamus, entorhinal and prefrontal cortices (Mehran et al., 2012). Interestingly, recent reports show insulin expression and production also in primary cultured astrocytes, which was decreased by amyloid-β (Aβ) and lipopolysaccharide (LPS) (Takano et al., 2018). Another putative source of brain-derived insulin may be the choroid plexus (Lamotte et al., 2004; Yong et al., 2011).

Regardless of its origin, it is clear that insulin has different effects on brain function and may play a crucial role in some pathological conditions.

## Central Actions of Insulin

Insulin primarily plays a role in the regulation of glucose uptake of insulin-sensitive cells, with its effect on peripheral tissues such as muscle, adipose tissue, and liver, being very similar. Activation of its receptor leads to phosphorylation and activation of AKT and ERK pathways, culminating in the mobilization of glucose transporter 4 (GLUT4) to the cell membrane, allowing greater glucose uptake by these cells. The brain, however, behaves in a very different way, mainly expressing the insulin-insensitive glucose transporters GLUT1 (in astrocytes and blood-brain barrier endothelial cells) and GLUT3 (in neurons). Consequently, classical modeling of glucose uptake by cells in the brain considers this to be an insulin-insensitive process, although this is subject to some controversy, as indicated above. In contrast, central insulin effects are primarily regarded as neurotrophic, affecting synaptic physiology and, hence, memory and learning.

Insulin and its receptor have been implicated in neurite outgrowth and axon guidance, through activation of the PI3K/AKT pathway, as demonstrated in Drosophila (Song et al., 2003; Gu et al., 2014), murine (Grote et al., 2011) and human neuronal cells (Liu et al., 2013; Roloff et al., 2015). IRS p53 seems to play an essential role in dendritic arborization. IRSp53 is expressed in the post-synaptic membrane of neurons, where it co-localizes with the post-synaptic density and interacts with proteins that constitute the cytoskeleton (Abbott et al., 1999; Chiu and Cline, 2010). Overexpression of IRSp53 in neuronal cultures had been shown to correlate with higher levels of arborization (Govind et al., 2001), while its inhibition reduces the density and size of dendritic spines (Choi, 2005).

Insulin can modulate synaptic activity and plasticity by several different mechanisms, inducing the endocytosis of AMPA receptors for the generation of long-term depression in hippocampal cell cultures (Beattie et al., 2000) and the modulation of NMDA receptors in the post-synaptic membrane, linked to synaptic strengthening (Skeberdis et al., 2001). The modulation of these glutamatergic receptors allows insulin to participate in neuronal activity-dependent synaptic plasticity (Van Der Heide et al., 2005). Overall, such data clearly links central insulin effects to neuronal plasticity processes underpinning cognitive functioning.

## Insulin Signalling and Autophagy in Neurodegerative Diseases: an Introduction

**FIGURE 2:**
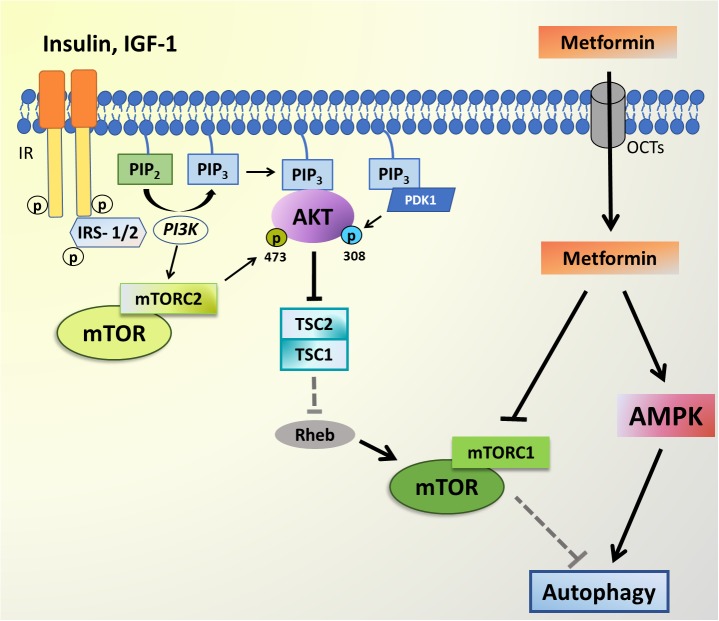
PI3K-AKT-mTOR signaling and Metformin targets. The AKT signaling pathway can be triggered by insulin-like growth factor 1 (IGF-1) and insulin, which bind to transmembrane receptors tirosine kinase (RTK) leading to IRS-1/2 phosphorylation, PI3K activation and subsequent phosphatidylinositol-4,5-bisphosphate (PIP_2_) to phosphatidylinositol-3,4,5-trisphosphate (PIP_3_) conversion. AKT and PDK1 bind to PIP_3_ at the plasma membrane, and PDK1 phosphorylates the activation loop of AKT at T308. RTK signaling also activates mTOR complex 2 (mTORC2) through an unknown mechanism, and mTORC2 phosphorylates AKT on S473. Active AKT is responsible for the inhibition of negative regulators of mTOR, such as tuberous sclerosis complex (TSC1/2), which induces the mTOR activator Rheb, thereby releasing the mTOR complex to phosphorylate its targets. mTORC1 activity inhibits autophagy. The Metformin enters the cell through organic cation transporters (OCTs) and is able to inhibit mTORC1 complex, while also activating AMPK thus leading to autophagy activation.

Although the literature data is still conflicting, as revised by Rotermund et al. (2018), the use of Metformin, one of the most famous anti-diabetic drugs, demonstrated to have some positive effects in, for example, PD and AD animal models (Lennox et al., 2014; Patil et al., 2014; Lu et al., 2016; Katila et al., 2017). According to the literature, both acute and chronic Metformin administration showed to increase the levels of glucagon-like peptide-1 (GLP-1), an incretin known as an inducer of insulin secretion (Maida et al., 2011), that may lead to the activation of PI3K/AKT signaling and higher brain ATG7 levels, thereby promoting autophagy (Candeias et al., 2017). Besides that, Metformin acts by regulating the AMPK signaling, which is already associated with insulin resistance in T2D (Xu et al., 2012) and is also related with the autophagy process (Figure 2).

As demonstrated by Yamamoto et al. (2018), transient autophagy inhibition can rescue glucose intolerance in Becn1^F121A^ knocking mice fed with high-fat diet, which presents a constitutively active autophagy process, suggesting that finding the right regulation of autophagy may be a potential target for drug development for neurodegenerative diseases. Besides that, abnormal protein aggregates, which have been classically linked to neurodegenerative disorders (Lasagna-Reeves et al., 2011; Larson and Lesné, 2012; Lashuel et al., 2013) are known to be substrates of the ubiquitin-proteasome and macroautophagy systems (Filimonenko et al., 2010) that are less active along aging (Shibata et al., 2006). However, the relationship between these aggregates and cellular toxicity as well as between total plaque load and cognitive status remains a point of debate (Koss et al., 2016). Thus, this section summarizes and discusses the relationship between insulin signaling and autophagy in each of the neurodegenerative diseases.

## Alzheimer’s Disease (AD)

Some research associating cerebral insulin resistance and neurodegeneration indicates that alterations in cerebral insulin signaling may be a different and more complex process than processes derived from classical type I and II diabetes pathophysiology (Rivera et al., 2005; Steen et al., 2005). As indicated above, both *in vitro* and *in vivo* studies show insulin effects in neuronal cells, specifically on synaptic plasticity as well as its positive regulation of memory and learning. The positive effects of insulin in learning were first shown in 1976 when insulin administration attenuated memory impairment in rats subjected to hippocampal lesions (de Castro and Balagura, 1976). This was followed by similar work showing insulin to positively regulate spatial learning and memory post-ischemia, while also reducing neuronal necrotic tissue in the hippocampal CA1 region (Voll et al., 1989). Insulin signaling through its receptor in the pyramidal neurons of the CA1 is also implicated in spatial learning in the water maze test (Zhao et al., 1999, 2004). Intracerebroventricular injection of insulin also improves memory, as tested by a passive-avoidance test (Park et al., 2000).

In recent decades, insulin effects on human cognition have been described, including effects on selective attention and verbal memory in healthy participants within hours of insulin infusion (Kern et al., 2001). Given that intravenous infusions of insulin produce a range of unwanted effects, such as hypoglycemia and blood ionic imbalance, different routes of administration had to be used in order to achieve a brain-specific action. It was known for more than a decade that peptides and small proteins could access the CSF from the nasal mucosa through the intercellular spaces in the olfactory nerve and olfactory bulb (Balin et al., 1986; Sakane et al., 1991; Illum, 2000). This prompted Benedict et al. (2004) to investigate the effects of intranasal insulin on the memory of 38 healthy participants over an 8-week period (Benedict et al., 2004), with results showing improved word-recall memory scores, mood assessments, and self-confidence. Importantly, these effects were achieved without any disturbances in blood glucose or insulin levels. Hence, intranasal insulin has become the preferred route of insulin administration for studies investigating insulin effects in the brain.

Data in recent years have suggested a direct relationship between autophagic processes and the development of type II diabetes. In 2013, He et al. (2013) showed that Beclin 2, like Beclin 1, is an important modulator of autophagy. Beclin 2 heterozygous-deficient mice (beclin2^+/-^) were fed for 8 weeks with a regular or high-fat diet (60% fat) with results in either condition showing an increase in body weight, food intake and in insulin resistance (He et al., 2013). Another study performed in diabetic and non-diabetic C57BL/6 mice observed an increase in autophagic vacuole formation in the presence of insulin resistance. The selectively inactivation of ATG7 in pancreatic β-cells showed that in the absence of autophagic process, ubiquitinated proteins and damaged organelles accumulate over time leading to pancreatic β-cell toxicity (Ebato et al., 2008).

Constitutively active autophagy can be achieved via a point mutation in Becn 1/Beclin 1 (Becn1^F121A/F121A^). With a high-fat-diet challenge, such autophagy-hyperactive mice show improved insulin sensitivity dispite an increase in degradation of insulin granules in pancreatic β-cells (Yamamoto et al., 2018). The crossing of Becn1^F121A^ mice with a transgenic murine model of AD, (5 × FAD), showed autophagy hyperactivation to significantly decrease Aβ accumulation, and to prevent cognitive decline (Rocchi et al., 2017). Autophagy hyperactivation in the 5 × FAD Becn1^F121A^ murine model leads to lower levels of both soluble and insoluble Aβ42 in both the cortex and hippocampus, whilst no changes in amyloid precursor protein (APP) levels were observed. In a cellular model using HEK293 cells expressing Becn1^F121A^ and APP, cells were transfected with siRNA to knockdown ATG7, leading to an increase in Aβ42, indicating the importance of autophagy in the regulation of Aβ formation (Rocchi et al., 2017). This suggests that strategies to hyperactivate the autophagic process, such as pharmacological treatment with ML246 or voluntary exercise, could decrease the risk of AD and cognitive decline (Rocchi et al., 2017).

In rodents, Caccamo et al. (2010) observed that 3 × Tg-AD mice fed for 10 weeks with rapamycin-containing food, versus a control diet, had a better performance in the Morris Water Maze task, suggesting a role for rapamycin in rescuing mice from early learning and memory deficits. 8 months of chronic rapamycin administration decreases mTOR activity in association with a decrease in hippocampal Aβ accumulation and tau hyperphosphorylation. Interestingly, this produced an autophagy gain of function, as indicated by raised levels of ATG7, ATG5/ATG12 complex and LC3-II (necessary of autophagy induction) in rapamycin-treated versus control 3 × Tg-AD mice. This effect was prevented by an autophagy inhibitor, 3-methyladenine (Caccamo et al., 2010).

Analyzing and comparing postmortem brain inferior parietal lobule tissue from patients diagnosed with mild cognitive impairment (MCI), AD, and healthy matched controls, Tramutola et al. (2015) showed that an increase in AKT and PI3K (p85 subunit) phosphorylation occurs in MCI and AD patients, coupled with mTOR hyperactivation. This increase in PI3K-AKT-mTOR signaling pathway was observed in early stage MCI as well as increased phosphorylation in the inhibitory site of IRS-1, coupled to a decrease in the autophagy markers, Beclin 1 and LC3, suggesting that such changes underpin the association of insulin resistance to early dementia. Importantly, the decrease in autophagy markers directly correlated with an increased Aβ levels. In a group of pre-clinical AD patients, with normal cognitive function ante-mortem coupled to AD neuropathology at autopsy, there was evidence of autophagy impairment although no changes in the PI3K-AKT-mTOR pathway were found, suggesting that disrupted autophagy may be an early event in Aβ deposition (Tramutola et al., 2015).

In a postmortem analysis of the midfrontal cortex gray matter, Beclin 1 levels were significantly decreased in severe AD (-30%) and mild-cognitive impairment (MCI) (-70%), compared to healthy controls. However, no significant decrease was observed in Huntington’s disease (HD) or the LB variant of AD patients (Pickford et al., 2008). The same study also investigated cortical Beclin 1 levels in two different lines of aging APP transgenic mice expressing extensive Aβ deposits and high levels of mutant human APP, finding that Beclin 1 expression was the same as in nontransgenic, age-matched littermate control mice. Such data would suggest that autophagy impairment could happen in upstream events before APP pathology (Pickford et al., 2008). However, it should be noted that direct evidence of APP degradation by autophagic processes is the subject of some controversy (Boland et al., 2010; Jaeger et al., 2010; Tian et al., 2013; Rocchi et al., 2017).

**FIGURE 3:**
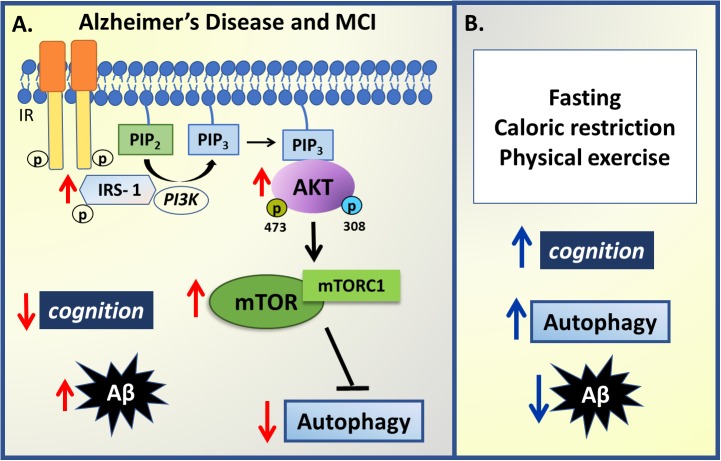
Summary of insulin –autophagy signaling changes in Alzheimer’s disease. **(A)** In mild cognitive impairment (MCI) and Alzheimer’s Disease, an inverse correlation between cognition levels, insulin sensitivity, autophagy and amyloid-β (Aβ) aggregates is observed. An increase in IRS-1, PI3K and AKT phosphorylation coupled to mTORC1 hyperactivation downregulates autophagy activity, impairing Aβ degradation, which results in increased Aβ deposition and decreased cognition performance. **(B)** However, some non-pharmacological strategies have been presented to modulate some changes observed in AD and MCI such as fasting protocols, caloric restriction and physical exercise that can improve cognitive performance, increase autophagy and thus decrease Aβ aggregates. Red arrows represent changes in pathophysiology and blue arrows represent changes promoted by non-pharmacological strategies.

Several processes and behaviors can regulate autophagy, including calorie restriction and exercise, which all improve insulin signaling and slow neurodegenerative processes. In rodents and primates maintained for several months on alternate day fasting, neurons are protected from neurodegenerative processes, in association with higher levels of brain derived neurotrophic factor (BDNF), CREB, and autophagy, as well as lower mTOR levels, improved mitochondrial function and decreased oxidative stress (reviewed in Mattson et al., 2017). Preclinical data indicate that intermittent fasting and alternate day fasting, which increase autophagy-promoting sirtuins, afford neuronal protection even in the presence of Aβ aggregates and tau accumulation, resulting in protection against cognitive impairment in 3 × TgAD mice (Halagappa et al., 2007). The mechanisms underlying the autophagy-promoting benefits of such calorie restriction diets require clarification, as these mechanisms are likely to afford some protection against neurodegenerative processes, especially in their early stages. Of note, a 24-hour fasting has been shown to increase autophagosomes and decrease mTOR signaling (Alirezaei et al., 2010). In the 5 × FAD transgenic mice that express mutant human APP770 and develop high levels of Aβ deposition at 3 months age, 48 hours of fasting was able to increase autophagy, although insufficient to clear the high levels of Aβ present in this model (Chen et al., 2015). As indicated previously, exercise is a significant positive regulator of autophagy, Aβ clearance, and improved cognition (Rocchi et al., 2017), with aerobic exercise, as well as food restriction, having a positive impact on cognitive performance of healthy participants (reviewed in Camandola and Mattson, 2017) and in animal models of AD (García-Mesa et al., 2011; Figure 3).

## Parkinson’s Disease

PD is a neurodegenerative disease with a slow progress that seems to begin many years before diagnosis with non-motor features such as olfactory and autonomic dysfunction, intestinal dysregulation, sleep disorders, fatigue, cognitive impairment and psychiatric symptoms. With disease progression classical motor symptoms appear, including bradykinesia, rest tremor, muscular rigidity and postural impairment. Risk factors include genetic and environmental factors with classical symptomatology mediated by the loss of dopaminergic neurons within the substantia nigra pars compacta (Snpc), although other neuroanatomical regions can be also affected (Kalia and Lang, 2015).

In PD, as in many other neurodegenerative diseases, an increase in intracellular protein aggregates, LB, composed of abnormal aggregates αSynuclein protein (αSyn), is evident. Although the relationship between LB pathology and PD pathoetiology still requires clarification, there is epidemiological evidence to indicate that insulin signaling impairment is a relevant modulator (Driver et al., 2008; Schernhammer et al., 2011).

A study of 110 PD patients (53 with PD and dementia (PD/D) and 57 PD without dementia evaluated glucose and insulin levels after a 2 hours oral glucose tolerance-test (Bosco et al., 2012). These authors found that PD/D patients had higher prevalence of insulin resistance, with abnormal glucose metabolism (Bosco et al., 2012), although it should be noted that the association of diabetes with PD is complex and controversial (reviewed in Santiago and Potashkin, 2013). However, recent conceptualizations of type 2 diabetes have placed an emphasis on the importance of the gut-liver-pancreas axis, with alterations in the gut long appreciated as an early pathophysiological symptom in PD (Parkinson, 2002) as well as a possible site for the vagal transport of αSyn to the brain (Anderson et al., 2016). Clearly such systemic changes require further investigation.

In a postmortem study of four PD patients’ brains homogenates from the substantia nigra and their age-matched controls, Sekar and Taghibiglou (2018) observed a significant decrease in PI3K p85, AKT, PIP_3_, IRS-1 and IR levels, as well as an increase in glycogen synthase kinase (GSK)3β and nuclear translocation of tumor suppressor phosphatase and tensin homolog deleted on chromosome ten (PTEN), strongly suggesting an association between PD and insulin resistance (Sekar and Taghibiglou, 2018). Elevated levels of phosphorylated IRS-1 on serine residues block insulin/IGF-1 binding to the IR and so down-regulate downstream effectors such as AKT, which in turn blocks GSK3β phosphorylation. Once dephosphorylated, GSK3β becomes active and phosphorylates tau protein leading to hyperphosphorylation and neurofibrillary tangles deposition (Athauda and Foltynie, 2016). In advanced stages of AD and PD, protein aggregates of tau, Aβ and αSyn coexist (Yang et al., 2018).

In PD disruption of autophagy seems to be a process that is at least partially independent of alterations in the PI3K/AKT/mTOR pathway (Heras-Sandoval et al., 2014). Data from literature have been shown that overexpression of αSyn inhibits autophagy in cell culture and *in vivo* leading to accumulation of aggregate-prone proteins (Winslow et al., 2010). The αSyn aggregates can be alleviated by increased expression of Beclin 1, as observed in brain of transgenic mice model of αSyn overexpression, causing increased autophagy and neuronal protection (Spencer et al., 2010). Furthermore, work by Dehay et al. (2010) on a human neuroblastoma cell line as well as in the 1-methyl-4-phenyl-1, 2, 3, 6- tetrahydropyridine (MPTP) mouse model of PD led the authors to propose that the increased mitochondrial reactive oxygen species (ROS) evident in PD leads to abnormal permeabilization of lysosomal membranes, thereby causing lysosomal depletion and the accumulation of autophagosomes, resulting in inefficient autophagy (Dehay et al., 2010). The role of oxidative stress-induced disruption in autophagy was also confirmed by other studies (Lin and Kuang, 2014; Navarro-Yepes et al., 2014; Filomeni et al., 2015; Teves et al., 2018). An increase in autophagic flux was also measured in lysates from a dopaminergic neuroblastoma cell line (SH-SY5Y) and mouse embryonic fibroblast cells treated with 6-hydroxydopamine (6-OHDA) for 3 hours, which was coupled to a reduction in glutathione content, indicating that treatment with 6-OHDA leads to an increase in ROS levels, and AMPK/ULK1 activating autophagy, in an mTOR independent pathway (Urano et al., 2018).

Furthermore, increasing evidence has implicated lysosomal dysfunction in PD as an important factor in autophagy disruption. It should be noted that a new type of nonfibrillar phosphorylated αSyn (pαSyn^∗^), which is conformationally distinct, has been identified in PD patients’ brains, which seem to be the result of an incomplete autophagic degradation of pαSyn. The pαSyn^∗^ associates with mitochondria and is highly mitotoxic, causing structural damage. According to the author, pαSyn^∗^ may be the missing link between LB and mitochondrial impairment (Grassi et al., 2018). Furthermore, Grassi et al. (2018) propose that preformed pαSyn fibrils activate autophagy but the incomplete and/or abnormal autophagolysosomal degradation observed in PD is responsible for generation of pαSyn^∗^ that should migrate away from the fibrils and associate with mitochondria. The incomplete autophagic degradation can also be caused by mutations in ATP13A2 gene and SYT11, both observed in PD (Bento et al., 2016). This is an area clearly requiring further research.

Regarding insulin signaling in humans, a recent PD trial tested the effect of Exenatide, the first synthetic agonist of glucagon like peptide 1 (GLP-1), indicated to restore insulin pathway in type II diabetes (Sandoval and Sisley, 2015), and previously known to preserve mitochondrial function in dopaminergic neurons, reducing apoptotic death and lysosomal depletion as well as decreasing tau hyperphosphorylation and plaque load (reviewed in Athauda and Foltynie, 2018). The Exanatide-PD trial analyzed 60 idiopathic PD patients (both genders) who received exenatide or placebo, once weekly for 48 weeks, followed by a withdrawal of 12 weeks for analysis. Extracellular vesicles isolated from blood samples were used to indicate the brain insulin signaling pathway. Results showed Exenatide-treated patients to have a sustained increase in IRS-1 phosphorylation levels, total AKT and pAKT S473 after 24 weeks treatment and an increase in total and p-mTOR (S2448-mTORC1) at 48 weeks of treatment, compared to placebo. Increased levels of total mTOR (mTORC1/2) were associated with sustained clinical improvement on motor scores at 60 weeks (Athauda et al., 2019).

On the other hand, effects of rapamycin, a specific inhibitor of mTOR, require further clarification in PD models. In the 6-OHDA rat model of PD, Jiang et al. (2013), observed that rapamycin treatment with three different doses was able to decrease oxidative stress and mitochondrial injury consequently avoiding apoptotic dopaminergic neuronal loss (Jiang et al., 2013). These effects involved modulation of the mTORC1 complex, as mTORC2 is unresponsive to acute rapamycin treatment (Perluigi et al., 2015). Another study using the 6-OHDA model showed mTORC1 inhibition by rapamycin, or the selective blockade of its downstream target, S6K (by PF-4708671), to improve the memory deficit evident in this model. Rapamycin treatment was also able to reduce the anxiety and depression-like behavior observed in the rat PD model (Masini et al., 2018).

Non-pharmacological strategies to improve PD symptoms indicate exercise to have beneficial effects. In the 6-OHDA model, 8 weeks of 4 days/week treadmill running protected animals from 6-OHDA-induced oxidative stress (Tuon et al., 2012), whilst in the MPTP murine model, 8 weeks of treadmill running, 5 days/week, reduced αSyn levels in the striatum, and dopaminergic neuronal loss, as well as improving motor deficits and recovery of Beclin-1 and LC3-II levels, indicating that exercise could up-regulate autophagy (Koo and Cho, 2017). It is important to note that exercise duration seems to be a critical factor for the benefits observed in animals, as shorter periods of exercise are not so effective (Aguiar et al., 2014).

However, in human studies the effects of exercise in PD patients are much less conclusive (Ebersbach, 2015), although a large cohort study published by National Parkinson Foundation Quality Improvement Initiative Database in 2014 showed that a higher frequency on exercise leads to lower levels of disease severity and slows cognitive decline. The study analyzed data from 4,866 PD patients who were at different disease stages, although all were receiving treatment in United States centers of excellence (Oguh et al., 2014). Whether any exercise benefits are evident at later stages of PD still requires determination (Petzinger et al., 2013).

Other factors known to modulate autophagy, including dietary restriction, may also slow PD progression, as indicated in the MPTP rhesus monkey model, as indicated by the numbers of nigro-striatal dopaminergic neurons (Maswood et al., 2004). Any strategy that inhibits the insulin-AKT-mTORC1 signaling pathway is associated with increased longevity via autophagy activation (Ntsapi and Loos, 2016), and therefore would be expected to positively modulate PD incidence and pathophysiology (reviewed in Mattson, 2010). The benefits of dietary restriction in PD patients remain to be determined.

**FIGURE 4:**
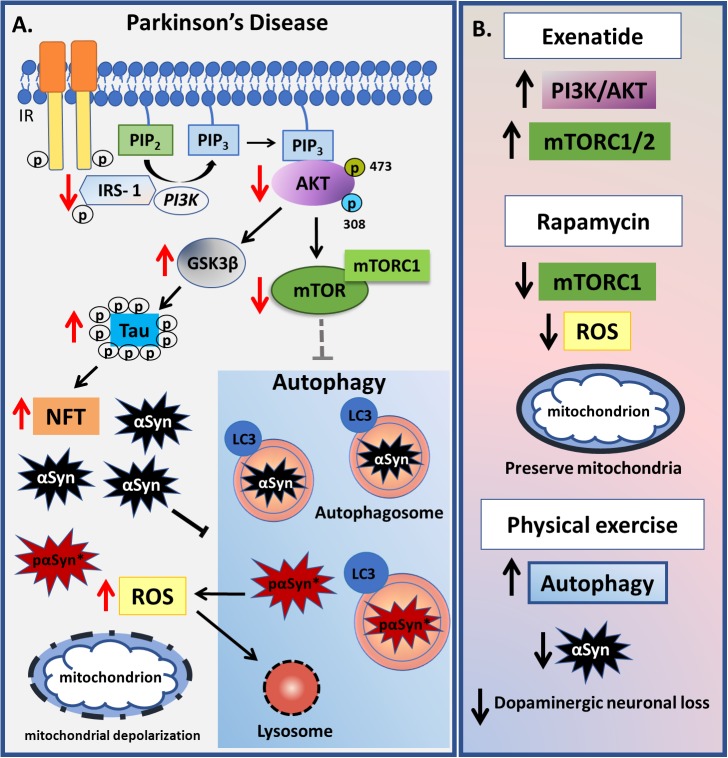
Summary of changes related to insulin-autophagy pathway observed in Parkinson’s Disease. **(A)** In Parkinson’s disease, a decrease in PI3K-AKT-mTOR signaling is observed, which leads to GSK3β activation. GSK3β hyperphosphorylates tau protein leading to neurofibrillary tangles deposition. mTORC1 complex is inactive which causes autophagy to become activated. Otherwise, high levels of α-Synuclein (αSyn) impair the autophagic process together with increased mitochondrial reactive oxygen species (ROS). ROS leads to abnormal permeabilization of lysosomal membranes, thereby causing lysosomal depletion and the accumulation of autophagosomes, resulting in inefficient autophagy. As a result of an incomplete αSyn autophagic degradation, a new reactive specie called pαSyn^∗^ is generated inside autophagosomes and when released in cytosol it associates with mitochondrion being highly mitotoxic. Damaged mitochondrion is indicated by dashed line. **(B)** Some pharmacological and non-pharmacological strategies that act in PI3K-AKT-mTOR-autophagy signaling are presented. Exenatide-treated patients have a sustained increase in IRS-1 phosphorylation levels, total AKT and pAKT as well as increased levels of mTOR, reverting the impaired signaling observed in PD pathophysiology. Rapamycin, which specifically blocks mTORC1 activity, was able to decrease oxidative stress and mitochondrial injury in animal models, whereas physical exercise is associated with an increase in autophagy and a decrease in both αSyn aggregates and dopaminergic neuronal loss.

In summary, correlations of insulin resistance with abnormal autophagy seem important processes in the pathogenesis of AD and PD. However, there are some important differences across these disorders that future research should clarify. Clearly, increasing autophagic processes is beneficial in AD, whilst this requires further studies in PD patients, including as to how concurrent dementia interacts with wider PD processes, given that PD patients with dementia are more prone to develop insulin resistance. PD patients also have lower levels of the IR and its downstream proteins, although the origin of autophagy abnormalities remains undetermined. The roles of autophagy and mTOR in PD are still highly controversial, which considerably complicates treatment aimed at such processes and pathways (Figure 4).

## Huntington’s Disease

Huntington’s disease is an autosomal dominant disorder characterized by severe motor and cognitive dysfunction, psychiatric disturbances and, ultimately, death. HD has long been appreciated to arise from a mutation in the Huntington (Htt) gene, which has a cytosine-adenine-guanine (CAG) expansion that encodes a polyQ repeat at the N-terminus in HD, resulting in impairments in protein functions and in protein aggregates (MacDonald et al., 1993). There is still no specific treatment for this condition, although studies in HD murine models show that the abolition of the expression of mutant Htt (mHtt) can rescue some of the symptoms and decrease protein aggregation in the brain (Yamamoto et al., 2000; Harper et al., 2005; Kordasiewicz et al., 2012). However, as functional Htt protein plays an important role in several physiological processes, such as mitochondrial dynamics, vesicle trafficking, axonal transport, synaptic function, and anti-apoptotic activity as well as autophagy, there are major problems in trying to suppress the activity of this gene (Choi et al., 2014; Ismailoglu et al., 2014; Wong and Holzbaur, 2014; Weiss and Littleton, 2016).

The loss of Htt functioning results in autophagy dysregulation, leading to protein accumulation and is likely to contribute to HD pathogenesis (Gelman et al., 2015). In contrast to other protein aggregation diseases, human and rodent HD samples show an upregulation of autophagosomes (Kegel et al., 2000; Petersen, 2001). However, these autophagosomes show some deficits in cargo recognition, resulting in inadequate degradation of aggregate proteins and damaged organelles (Boland et al., 2008; Martinez-Vicente et al., 2010). Besides that, it is also known that the silencing of either Htt or its interactor, Htt-associated protein 1 (HAP1), impairs the transport of autophagosome along the axon (Wong and Holzbaur, 2014), indicating that mHtt also modulates endocytic trafficking.

According with the literature, HD patients present higher basal plasma levels of IGF-1, which have been linked to the cognitive decline seen in these patients, leading to the hypothesis that IGF-1 resistance may be one of the underlying mechanisms involved in this pathology (Saleh et al., 2010; Salem et al., 2016). IGF-1, an upstream activator of the AKT-mTOR pathway, can inhibit mHtt-induced neuronal death and decrease the formation of intranuclear mHtt inclusions, via AKT phosphorylation of Htt, thereby providing a neuroprotective effect in HD (Humbert et al., 2002). Besides that, data from HD animal model showed AKT to be up-regulated with the intranasal administration of recombinant human IGF-1, increasing phosphorylation of mutant Htt and leading to an improvement in motor activity, as well as in both peripheral and central metabolic abnormalities (Lopes et al., 2014). On the other hand, according to Yamamoto et al. (2006), the dose-dependent clearance of the protein aggregate after insulin and IGF-1 treatment involves IRS-2 activation, and not IRS-1 or AKT activity (Yamamoto et al., 2006). Interestingly, depletion of the IGF-1 receptor (IGF-1R) resulted in decreased autophagy and increased mHtt levels, which was rescued only by prolonged (24 hours) IGF-1 treatment, in an AKT-independent pathway (Renna et al., 2013). Although another study demonstrated that IGF-1R deficiency affected only the size but not the mHtt levels in HD mice, it was observed that this deficiency had different consequences depending on the sex of the animal, causing some detrimental effects in the males, such as worsened rotarod performance and weight loss, but a delayed tremor onset in the females (Corrochano et al., 2014). Besides that, viral overexpression of the transcription factor EB (TFEB), a substrate of mTORC1 activity, resulted in increased autophagy and decreased levels of mHtt (Vodicka et al., 2016), indicating that the PI3K-AKT-mTOR pathway also plays an important role in this disease (Wu et al., 2009). As such, activation of the IGF-1/insulin pathway seems to have a dual and symptomatic utility, although the mechanisms involved require clarification.

**FIGURE 5:**
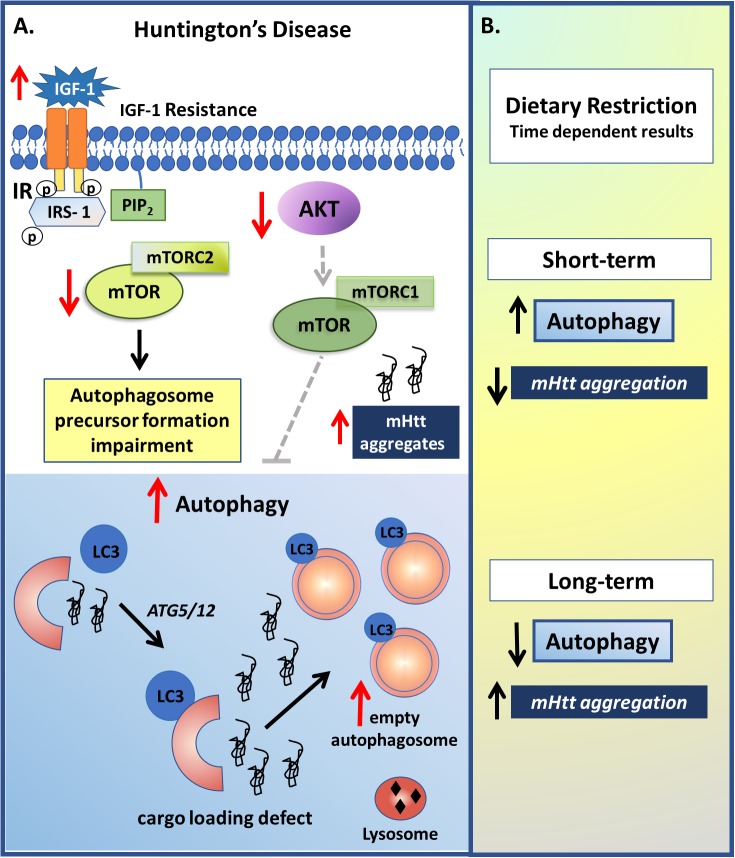
Insulin and autophagy signaling modulation in Huntington’s disease (HD). **(A)** Although the IGF-1 role on HD still seems paradoxical, it is clear that it develops an important role in this pathology. As observed in some studies, HD patients present higher basal plasma levels of IGF-1, which may be related with an IGF-1 resistance condition and a reduction in the insulin pathway. The reduction in IR activity results in reduced mTORC2 and AKT-mTORC1 activities, which leads to, respectively, a reduction in autophagosome precursor formation and an increase in the autophagy process, which may be related with the upregulation of autophagosomes with deficits in cargo recognition. However, long term IR down regulation, due to for example, receptor depletion or long term IR inhibition, results in reduced autophagy, which may be due to a lack of essential autophagosome precursor formation, and increased mHtt aggregates. **(B)** Non-pharmacological approaches such as dietary restriction also demonstrated to have a dual effect depending on the time. Short-term fasting resulted in increased autophagy and reduction on mHtt aggregation, while long-term serum deprivation resulted in reduced autophagosome formation.

Given that nutrient-rich or starvation diets can, respectively, suppress or increase autophagy via serine/threonine kinase phosphorylation of mTOR (Kim et al., 2008; Wong et al., 2015), a number of studies have investigated the utilization of dietary interventions in HD. Although the accumulation of mutant protein can lead to mTOR-independent autophagy with the degradation of accumulated proteins differing from the degradation under conditions of starvation (Yamamoto et al., 2006), dietary restriction in a HD mouse model resulted in an increase in autophagy and a reduction in mHtt aggregate formation and the normalization of blood glucose regulation, thereby contributing to a slower disease progression and an extended lifespan (Duan et al., 2003; Ehrnhoefer et al., 2018). However, although scheduled feeding demonstrates to increase autophagy, long-term serum deprivation, such as 8 to 24 hours, results in reduced autophagosome formation (Renna et al., 2013), suggesting that the autophagy regulation is influenced by the nutrient starvation time (Figure 5). Clearly, more studies are required on relevant processes in this area, including as to how dietary restriction may afford benefit in HD.

## Amyotrophic Lateral Sclerosis and Frontotemporal Dementia

Amyotrophic lateral sclerosis is a neurodegenerative disease that is characterized by the degeneration of upper and lower motor neurons of the primary motor cortex and spinal cord, resulting in muscle weakness and eventual paralysis, leading to death (Al-Chalabi and Hardiman, 2013). Although the pathoetiology of ALS is unknown, it has a not uncommon co-occurrence with FTD, which is marked by focal atrophy of both frontal and anterior temporal lobes (Van Langenhove et al., 2012). Moreover, as ALS and FTD share genetic risk factors, it has been proposed that they represent a continuum of neurodegenerative disorders (Van Langenhove et al., 2012).

ALS is associated with hypermetabolic and dyslipidemia states (Dupuis et al., 2008; Seelen et al., 2014), with an inverse risk factor association with obesity and type 2 diabetes (Gallo et al., 2013; Kioumourtzoglou et al., 2015). ALS patients with hyperlipidemia have an increased life expectancy, suggesting a neuroprotective role of lipids on ALS pathophysiology (Dupuis et al., 2008). Similar results are evident in the SOD1 mutant murine model of ALS, where high energy-diets prolong the lifespan (Dupuis et al., 2004). However, after adjusting for markers of disease severity, such as body mass index (BMI), forced vital capacity, and age, it was found that BMI, but not dyslipidemia, underlie this increased survival of ALS patients (Paganoni et al., 2011).

Although the role of lipids in ALS requires clarification, there is greater certainty as to the higher prevalence of hypermetabolism in ALS patients, which is linked with a faster rate of functional decline and shorter survival (Steyn et al., 2018). Interestingly, hypermetabolism is associated with some genetic risk factors for ALS, although not for the C9ORf72 mutation (Steyn et al., 2018), which is the one most commonly associated with ALS-FTD (DeJesus-Hernandez et al., 2011). Remarkably, alterations in the C9ORf72 gene seem to have a dual function in autophagy regulation, with C9ORf72 positively regulating the initiation of autophagy by controlling the ULK1 complex, with its reduction resulting in autophagic dysfunction and protein aggregation (Webster et al., 2016). Other work shows the C9ORf72 gene to decrease autophagy through mTORC1 regulation (Ugolino et al., 2016). However, although Steyn et al. (2018) were not able to find any association between C9ORf72 and hypermetabolism, Liu et al. (2018) showed that loss of C9ORf72 gene function impairs lipid digestion and increases *de novo* fatty acid synthesis through dysregulated autophagic process under cell glucose starvation conditions (Liu et al., 2018). This could suggest that starvation or nutrient-rich conditions may play an important role in modulating C9ORf72 functions in autophagy.

Mutations leading to a loss-of-function in the TANK-binding kinase (TBK)1 phenotype also lead to an increased risk of developing ALS and FTD due to autophagy impairment (Cirulli et al., 2015; Freischmidt et al., 2015; Cui et al., 2018). Interestingly, a study performed on obese zucker rats provides evidence for the involvement of TBK1 in the mechanism of insulin resistance (Muñoz et al., 2009). This study shows that obese animals have increased hepatic TBK1 levels, leading to decreased IR activity due to Ser994 phosphorylation by TBK1 (Muñoz et al., 2009). Moreover, a high fat diet can positively modulate TBK1 gene expression in the liver and white adipose tissue (Chiang et al., 2009) whilst its accumulation in lipid rafts of hypothalamic neurons disrupts IR activation as well as AKT signaling, suggesting a potential role of TBK1 in brain insulin resistance (Delint-Ramirez et al., 2015). Overall, such data suggest that it is plausible that TBK1 may be one of the underlying mechanisms by which obesity is associated with a reduced risk of ALS and FTD.

**FIGURE 6:**
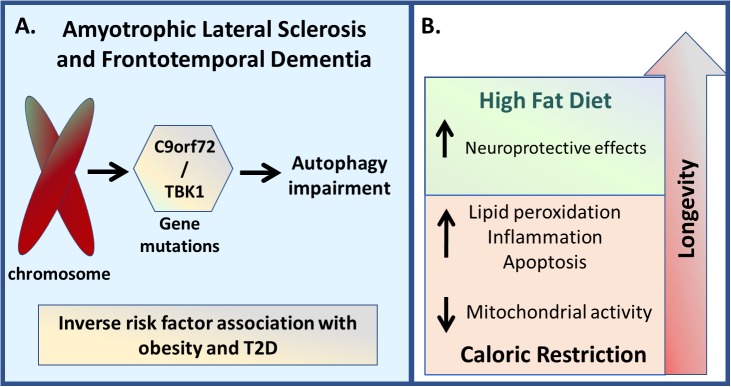
Amyotrophic lateral sclerosis and Frontotemporal Dementia. **(A)** Genetic variations in genes such as C9orf72 and TBK1 are linked with both ALS and FTD disease development, which are associated with autophagy impairment. Besides that, since it is also associated with hypermetabolic and dyslipemia states, both conditions seem to present an inverse risk factor association with obesity and T2D. **(B)** Regarding the non-pharmacological inhibition, caloric restriction demonstrated to decrease lifespan due to an increase in lipid peroxidation, inflammation, and apoptosis, as well due to a reduction in mitochondrial activity, while high-energy diets resulted in increased lifespan.

An animal model of ALS under caloric restriction has a decreased lifespan due to an increase in lipid peroxidation, inflammation and apoptosis, as well a decrease in mitochondrial bioenergetic efficiency, when compared with mice fed *ad libitum* (Patel et al., 2010). Similarly, rapamycin treatment is detrimental, increasing motor neuron degeneration in a mouse model of ALS (Zhang et al., 2011). As autophagy and metabolism modulate ALS disease onset and progression, dietary interventions may prove of clinical utility in the management of this disorder, including the possibility of using a high-fat diet as treatment (Figure 6).

## Other Neurodegenerative Diseases

### Spinocerebellar Ataxia Type 3

Spinocerebellar ataxia 3 (SCA3), a neurodegenerative disease characterized mainly by progressive ataxia affecting balance, gait and speech, is caused by a mutation in the C-terminus polyglutamine (PolyQ) region of the ataxin-3 protein, an ubiquitously expressed deubiquitinating enzyme with important functions in the proteasomal protein degradation pathway and regulation of transcription. According with the literature, the polyQ stretch in wild-type ataxin-3 induces autophagy by protecting Beclin-1 from proteasome-mediated degradation (Ashkenazi et al., 2017). As other neurodegenerative diseases, the only available treatments aim to reduce disease symptoms. However, although the underlying mechanisms are not completely understood, the regulation of autophagic process has demonstrated to be a potential target for ameliorating or stopping the progression of the disease (Menzies et al., 2010; Nascimento-Ferreira et al., 2011). As demonstrated by Menzies et al. (2010), the administration of rapamycin improved motor performance in an SCA3 animal model through mTOR inhibition and consequent autophagy upregulation. Besides that, according with Nascimento-Ferreira et al. (2011), Beclin-1 overexpression resulted in increased autophagic flux, mutant ataxin-3 clearance and other neuroprotective effects, rescuing the abnormal expression of endogenous autophagic markers, accumulation of autophagosomes and reduced levels of Beclin-1. In a similar way, another study observed decreased autophagosome production when comparing fibroblasts from SCA3 patients and healthy individuals. However, although Beclin-1 overexpression increased the autophagic flux, it did not result in higher levels of autophagosome production (Onofre et al., 2016). Taken together, this result supports the important role of autophagy in also regulating the SCA3 disease.

### Lafora Disease

Lafora disease is a rare autosomal recessive, progressive and lethal neurodegenerative disease that is characterized by myoclonic epilepsy followed by continuous neurological decline. Reported in approximately 58% of the LD cases, mutation in the EPM2A gene, which encodes the glucan phosphatase laforin, is associated with the presence of intracellular inclusion bodies known as Lafora bodies (LB) in the brain, spinal cord and other peripheral tissues (Knecht et al., 2010). Although laforin demonstrated not to be absolutely necessary for autophagy, it was observed that it positively regulates this process (Knecht et al., 2010). According to this study, LD is associated with decreased levels and impaired formation of LC3-II, as well altered signaling in the AKT-mTOR pathway, which was rescued after laforin overexpression, reducing the amount of protein aggregates in an autophagy-dependent manner (Knecht et al., 2010). Moreover, besides autophagy, LD also seems to be associated with insulin resistance and hyperglycemia (Nicolescu et al., 2019), strengthening the crosstalk between insuling and autophagy signaling in neurodegenerative diseases.

## Conclusion

Although it is clear that central insulin signaling and the regulation of autophagy are relevant to a host of diverse neurodegenerative disorders, which is supported by several studies including pharmacological inhibition, animal models, genetic strategies and patients, as well as by the link between metabolic disorders and the development of some neurodegenerative diseases, it is still hard to define statements as data from literature is controversial and the underlying mechanisms as well as the possible benefits of autophagy modulation seem to be specific for each disorder. As many key cellular processes are intimately associated with insulin signaling and autophagic process, pharmacological inhibition and/or stimulation may have some detrimental effects, especially considering long-term treatments, which may partly be responsible for the conflicting results in the literature. Besides that, it is important to consider that the onset of the disease, the age and also the sex can have a strong influence on the development and also on the treatment outcomes. In this context, genetic strategies that modulate metabolism and autophagy regulation can contribute to having a better understanding of the underlying mechanisms among these pathways and the neurodegenerative diseases. Besides that, as it seems that outcomes may vary according to stimulus duration, the study of the effects of non-pharmacological interventions such as physical exercise and intermittent fasting in the onset and progression of neurodegenerative diseases can also bring light to new information for the literature, as well as possible new approaches that may contribute to the conventional treatments. Thus, the understanding of how such processes are integrated in different central and systemic cells should provide better targeted treatment for these still poorly managed conditions.

## Author Contributions

CS and EK conceived the review idea, and edited final version of the text and figures. CM, NdM, and AO contributed equally writing the text. NdM and AO contributed equally conceiving the figures ideas. GdM contributed by drafiting the work and drawing the figures.

## Conflict of Interest Statement

The authors declare that the research was conducted in the absence of any commercial or financial relationships that could be construed as a potential conflict of interest.
